# Novel Approaches to Improve Iris Recognition System Performance Based on Local Quality Evaluation and Feature Fusion

**DOI:** 10.1155/2014/670934

**Published:** 2014-02-12

**Authors:** Ying Chen, Yuanning Liu, Xiaodong Zhu, Huiling Chen, Fei He, Yutong Pang

**Affiliations:** ^1^College of Computer Science and Technology, Jilin University, Changchun 130012, China; ^2^Key Laboratory of Symbolic Computation and Knowledge Engineering of Ministry of Education, Jilin University, Changchun 130012, China; ^3^College of Software, Nanchang Hangkong University, Nanchang 330063, China; ^4^College of Physics and Electronic Information, Wenzhou University, Zhejiang 325035, China

## Abstract

For building a new iris template, this paper proposes a strategy to fuse different portions of iris based on machine learning method to evaluate local quality of iris. There are three novelties compared to previous work. Firstly, the normalized segmented iris is divided into multitracks and then each track is estimated individually to analyze the recognition accuracy rate (RAR). Secondly, six local quality evaluation parameters are adopted to analyze texture information of each track. Besides, particle swarm optimization (PSO) is employed to get the weights of these evaluation parameters and corresponding weighted coefficients of different tracks. Finally, all tracks' information is fused according to the weights of different tracks. The experimental results based on subsets of three public and one private iris image databases demonstrate three contributions of this paper. (1) Our experimental results prove that partial iris image cannot completely replace the entire iris image for iris recognition system in several ways. (2) The proposed quality evaluation algorithm is a self-adaptive algorithm, and it can automatically optimize the parameters according to iris image samples' own characteristics. (3) Our feature information fusion strategy can effectively improve the performance of iris recognition system.

## 1. Introduction

Today, biometric recognition has become a common and reliable way to authenticate the identity of a living person based on physiological or behavioral characteristics. From birth to death, the pattern of the iris is relatively constant over a person's lifetime. Because of its uniqueness and stability, iris recognition is one of the most reliable human identification techniques. Currently, most iris recognition systems require a cooperative subject; however, capturing the entire iris may be infeasible in surveillance application. Therefore, partial iris recognition algorithms are going to play a significant role. Unfortunately, this will bring about two issues. (1) Whether partial iris image can effectively replace the entire iris image or not is still an open question. (2) At the same time, the way of selecting different regions of the iris texture information will influence the RAR because different iris regions contain different texture information. Broussard et al. [1] pointed out that the notable trait of partial iris recognition algorithms is the inner regions of iris which produce much less identification accuracy than the center or outer regions do; however, this does not indicate that inner regions of iris do not contribute to the accuracy of the entire template; it simply means that there is less stable, discriminatory information that existed in the inner iris regions.

In order to select the effective iris information, iris image quality evaluation is of great importance. Information from iris patterns is dispersed randomly and nonuniformly over the region of the iris image. Therefore, in order to solve the two problems mentioned above and maximize the RAR and stability of iris recognition system, it is more sensible to adopt local quality evaluation on different subregions rather than on entire iris image.

The rest of the paper is organized as follows.  Section 2 reviews the related work.  Section 3 analyzes different tracks' information.  Section 3 proposes the algorithm of fusion texture feature information of different tracks. Experiments design schemes and experimental results and discussion are presented in Sections 4 and 5, respectively.  Section 6 concludes this paper.

## 2. Related Work

Williams [2] pointed out that excellent enrollments and subsequent recognitions are obtained with 40% or less of the iris image available for analysis due to the excluded areas covered by eyelids, deep shadow, and specular reflection. Du et al. [3, 4] investigated the accuracy of using a partial iris image for identification and determined which portion of the iris has the most distinguishable patterns. However, there was no further analysis of the properties of different portions that have been provided in Du's experiments. Hollingsworth et al. [5] pointed out that not all the bits in an iris are equally useful. Comparing different regions of an iris to evaluate their relative consistency, they found that the middle bands of an iris are more consistent than the inner bands, and their conclusions differ from other researchers' findings. Yet, Hollingsworth has not given the experiments on the RAR of different portions. Pereira and Veiga [6] have considered that the information of iris patterns is dispersed randomly; therefore, they utilized genetic algorithm to find a distribution of points, which makes the iris recognition system get better results. But the flaw of this research is that no experiments were conducted on the RAR of different portions. The possibility that fragile bits exist was presented firstly by Bolle et al. [7], whose experimental results indicated that the invariant bits in the iriscode representation are dramatically robust to the imaging noise. On the basis of the previous works, Hollingsworth et al. [8] made use of some fragile bits rather than ignoring fragile bits completely. They used the coincidently fragile bit locations to improve the accuracy of matches and found that score fusion of fragile bit distance and Hamming distance work better than Hamming distance alone. Ma et al. [9] presented that the regions closer to the pupil provide the most useful texture information for recognition. So they extracted features only in the region of interest (ROI). Besides, Ma proposed an image quality assessment method by analyzing the frequency energy distribution of two subregions of an iris in the horizontal direction. However, Ma neither explained why the inner is better than the outer nor assessed the different subregions of the same iris image. Chen et al. [10] divided the iris area into multiple concentric bands and used continuous wavelet transformation to determine local quality evaluation measures for different regions. The quality score for the entire iris image is the weighted average of the band-wise local energy. Some experiments show that energy is a good indicator to distinguish iris features, and high values of energy indicate good quality. However, just using frequency domain transformation cannot completely reflect the texture information of different concentric bands. Yuan and Shi [11] divided the whole annular iris region into three virtual zones and extracted features in those three zones separately and differently according to the characteristics of their structures. But Yuan did not describe how to assign weighted values to different virtual zones. Tsai et al. [12] divided iris texture into three regions in the vertical direction, and further pointed out that the region closer to pupil usually contains more high-frequency components, the middle region consists of fewer and bigger irregular blocks and the region closer to limbic is usually covered with the eyelid and sparse patterns. Based on the theory of the importance of the iris features from different radial locations, they developed an encoded scheme to represent adjacent variations of the iris pattern and adopted a feature selection to select the significant feature. However, they just intuitively claimed that different regions have different information yet did not provide a theoretical and experimental proof. Broussard et al. [1] divided an iris into annuli and radial sectors to determine which portions of the iris show the best discrimination; they also have drawn six conclusions. But Broussard did not further process the different iris regions' feature. Poursaberi and Araabi [13] utilized the lower part of the encircled iris area for recognition; wavelet-based texture features were used in the process, and Hamming and harmonic mean distance were employed as classifier. Experiment results on CASIA database show that relying on a smaller but more reliable part of an iris improves the overall performance though the net amount of information is reduced. However, Poursaberi just concentrates on the ROI while without further describing the ROI selection criteria and the proportion of ROI.

We compared the pros and cons of some previous work (Table 1). In Table 1, the image database, feature extraction methods, evaluation methods, and their conclusions are summarized.

Based on the analysis shown in Table 1, it is reasonable to draw the conclusion that current researchers have not further dealt with the features of different regions. In the subsequent sections, following works are attempted to be done.

1. The well-known databases are taken as experimental sample databases. With new methods differing from that proposed in aforementioned literature, the normalized segmented iris is divided into several different numbers of tracks according to the image features of every iris database.
2. Multichannel 2D Gabor filters and gray level cooccurrence matrix (GLCM) are adopted for these tracks feature extraction; support vector machine (SVM) and k-nearest neighbor (KNN) are utilized for classification. By comparing different tracks' RAR, it is found that different tracks have different degrees of texture information.
3. In local quality evaluation on different tracks, evaluation parameters include local one-dimensional information entropy, local two-dimensional information entropy, local Haralick texture entropy, local Tamura texture measurement, and local gray variance. Meanwhile, in order to get the maximum of decidability between intraclass and interclass, PSO is used to optimize weighted coefficients.
4. Fusing different tracks' information according to different tracks' weighted coefficients to improve performance of iris recognition system.

## 3. The Proposed Method

### 3.1. Iris Image Preprocessing

**Figure 1:**
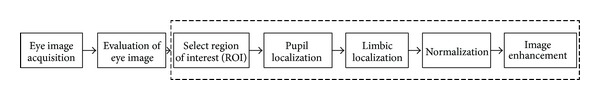
Diagram of image preprocessing.

Different iris localization algorithms are adopted for different iris image databases because these four iris image databases (CAISA-V1, CASIA-V3 Interval, MMU-V1, and JLUBRIRIS-V1) are of certain different features. Image of JLUBRIRIS-V1 is taken as sample to describe iris image preprocessing in detail. The specific process is shown in Figure 1.

**Figure 2:**
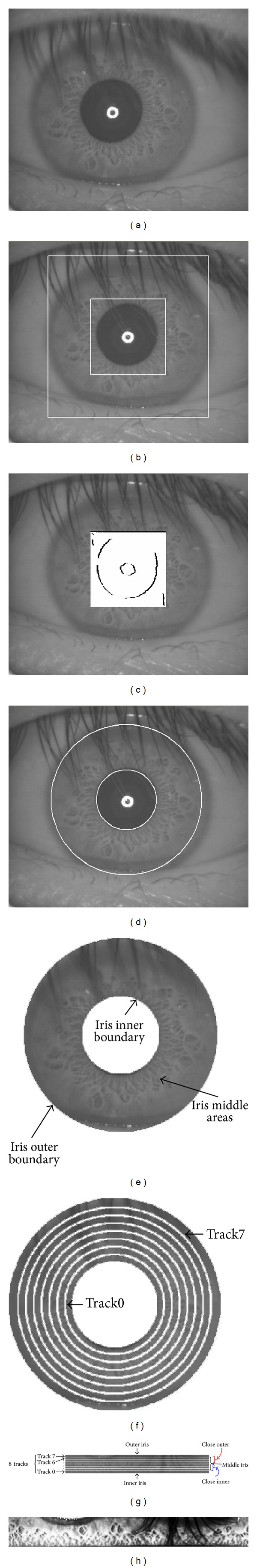
Steps involved in preprocessing. (a) Original iris image, (b) ROI selection, (c) Canny transformation, (d) pupil and limbic localization, (e) iris region segmentation, (f) iris region is divided into circular regions, (g) iris region is divided into 8 parts labeled with track0, track1,…, track7 from bottom to top, and (h) enhanced image after histogram equalization.

Firstly, poor quality images are filtered out through image quality evaluation. Secondly, two regions of interest are confirmed according to empirical values, and small ROI contained pupil area while big ROI contained iris area. Thirdly, locate the pupil area by means of Canny operator and Hough transform in the small ROI. Fourthly, locate the iris outer boundary by virtue of gray-level gradient difference in the big ROI. Fifthly, iris area is normalized by polar to Cartesian transformation [14]. Finally, global histogram enhancement is conducted on normalized image to eliminate the effects of external condition such as uneven illumination. The treatment affected diagrams during the image preprocessing are shown in Figure 2.

### 3.2. Analyze Different Tracks' Information with RAR

**Figure 3:**
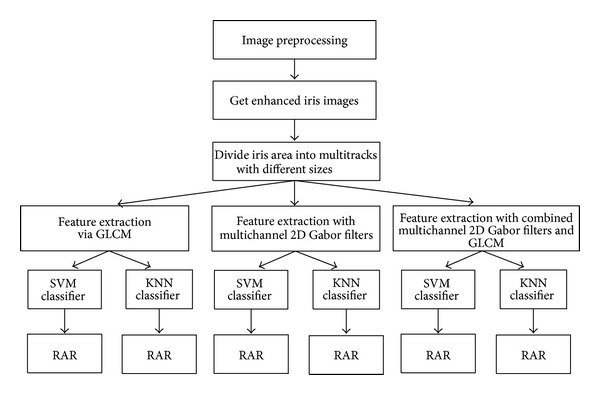
Analysis processing diagram.

Based on the analysis of the related work, the researchers of the present work attempted to analyze the influences of different tracks on RAR in detail. Analysis process is shown in Figure 3.

#### 3.2.1. Feature Extraction Methods

Multichannel 2D Gabor filters and GLCM are used for feature extraction. The former reflects the features of spatial transformation and frequency transformation, and the latter is a method based on statistics; the combined iris features are complementary features.

2D Gabor filters have the merits of tunable orientation, radial frequency bandwidths, and tunable center frequencies. Ma et al. [15] used multi-channel 2D Gabor filters to analyze the global feature. Nabti and Bouridane [16] utilized special Gabor filters for iris feature extraction. Multi-channel 2D Gabor filters are used to get texture information on various directions and different scales, each channel corresponding to a direction in a different scale. Gabor wavelet is obtained via conducting moderate-scale expansion and rotation on generating function *g*(*x*, *y*):

(1) gmn(x,y)=a−mg(x′,y′), a>1,{m,n ∣ m>1, n>1, m∈Z, n∈Z}.

For an image *I*(*x*, *y*), the Gabor transformation is defined as

(2) $$\begin{array}{c} f_{mn}\left(x,y\right)=\int I\left(x,y\right)g_{mn}^{*}\left(x-u,y-v\right)duv, \end{array}$$

where the asterisk denotes complex conjugate operation and (*u*, *v*) ∈ *Ω* is the size of filter window.

Finally, the iris feature vector (FS) representation is obtained by combining *μ*_*mn*_ with *σ*_*mn*_

(3) $$\begin{array}{c} \text{FS}=\left\{\mu_{00},\sigma_{00},\mu_{01},\sigma_{01},\ldots ,\mu_{M-1K-1},\sigma_{M-1K-1}\right\}, \end{array}$$

where *M* and *K*, respectively, denote the numbers of scales and orientations and *μ* and *σ* are the mean and standard deviations of transform coefficients, respectively.

GLCM, proposed by Haralick et al. [17], is one of the most prominent approaches, which used to extract textural features. The texture-context information is specified in a matrix of relative frequencies *P*_*ij*_ with two neighboring resolution cells. *P*_*ij*_ can be described by

(4) P(i,j ∣ d,θ)=#{((k,l),(m,n))∈(Lr×Lc)×(Lr×Lc) ∣ max⁡(|k−m|,|l−n|)=d, θ, I(k,l)=i, I(m,n)=j},

where *θ* and *d* are the directions and distances between two pixels in images, # denotes the number of elements in the set. Meanwhile, using GLCM to extract textures is sensitive to three factors, namely, window size, number of gray levels, and distances between pixel pairs. Traditionally, Haralick's features include fourteen features and the first twelve texture features are chosen in this study.

#### 3.2.2. Feature Classification Methods

SVM and KNN are introduced for classification. For more details about SVM, one can refer to [18], which provides a complete description of the SVM theory. In order to make the linear learning machine work well in nonlinear cases, the original input space can be mapped into some higher-dimensional feature space by using a kernel function. In this study, radial basis function (RBF) is used as kernel function.

As one of the supervised learning algorithms, KNN is a more widely used classifier because of its simplicity and efficiency. Sebastiani [19] pointed out that KNN defers the decision on how to generalize beyond the training data until each new query instance is encountered. For more details about KNN, we refer the reader to [19].

### 3.3. Quality Evaluation of Different Tracks

Iris image quality evaluation is generally classified into two kinds, namely, global and local evaluation. The former focuses on the entire image, but the latter focuses on a number of subimages of given image; we employ the latter in this study.

**Figure 4:**
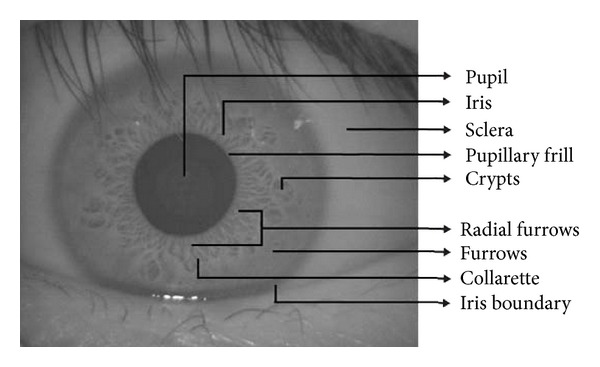
The complex textural patterns of image.

Daugman [14] employed quality metric by combining global evaluation with local analyses to measure defocus, motion, occlusion, and reliable iris code bits to control image acquisition and improve performance. Proenca [20] assessed the quality of visible wavelength light iris samples captured under unconstrained conditions according to focus, motion, angle, occlusions, area, papillary dilation, and levels of iris pigmentation. Kang and Park [21] proposed the hierarchical structure of three SVM classifiers to measure the iris size (IS), the amount of motion (AM), the visible iris region ratio (VIR), and the focus score (FS). The first SVM was used for IS and AM; the second SVM was used for VIR and FS; the output of the first two SVM were fed into the third SVM and final decision was made to indicate which image has better quality. Kalka et al. [22] proposed two-step quality assessment algorithm. The first step was to individually estimate the defocus blur, motion blur, off-angle, occlusion, lighting, specular reflection and corresponding pixel counts, and the second step was to fuse the estimated factors via a Dempster-Shafer theory approach. Belcher and Du [23] mainly focused on measuring feature information. They proposed an approach to select the portions of an iris with the most distinguishable changing patterns. The feature information score, the occlusion score, and the dilation score were fused to form quality score. However, Belcher and Du merely evaluated the whole normalized iris area instead of subiris. Zhu et al. [24] assessed image quality via analyzing the coefficients of the particular area of iris texture with the help of discrete wavelet decomposition. Wei et al. [25] adopted 2D Fourier spectrum for defocused images and simple gray statistical feature for motion and occlusion images. From the above analysis, it can be seen that most scholars focused on evaluating the entire iris image or normalized iris area instead of on different local tracks of normalized iris region. However, the iris texture shows abundant characteristic factors, and thereby iris feature extraction is an effective way to describe texture details. As shown in Figure 4, the iris image includes plenty of texture information such as contraction furrows, crypts, collarets, and radial furrows.

**Figure 5:**
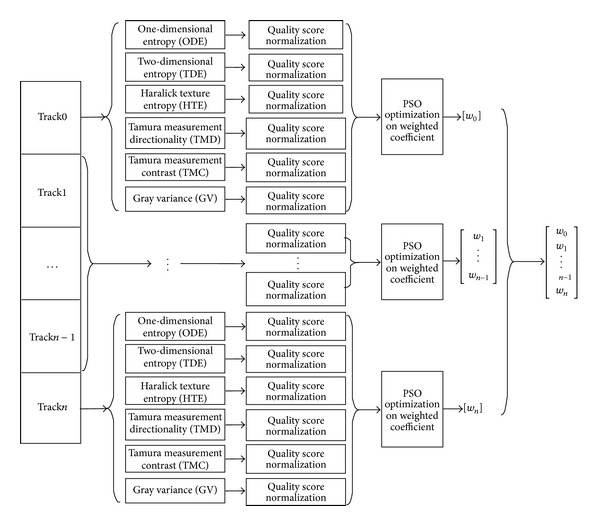
Quality evaluation block diagram.

This study focuses on evaluating iris image quality through evaluation of texture information degree because iris image has rich texture information. One-dimensional information entropy (ODE), two-dimensional information entropy (TDE), Haralick texture entropy (HTE), Tamura measurement directionality (TMD), Tamura measurement contrast (TMC), and gray variance (GV) are adopted as texture evaluation methods. To our best knowledge, these six evaluation methods have their own merits when used for evaluating image texture. The local quality evaluation block diagram is shown in Figure 5.

#### 3.3.1. Local One-Dimensional Information Entropy

The concept of information entropy describes how much information is provided by the signal or image. Entropy can be taken as a measure of the uncertainty of a random variable *X*. Let *X* be a discrete random variable with a finite alphabet set containing *N* symbols given by {*x*_0_, *x*_1_,…, *x*_*N*−1_}. If an output *x*_*i*_ occurs with probability *p*(*x*_*i*_), then the amount of information associated with the known occurrence of output *x*_*i*_ is defined as *I*(*x*_*i*_) = − log⁡_2_⁡*p*(*x*_*i*_); that is, for a discrete source, the information generated in selecting symbol *x*_*i*_ is ⌈−log⁡_2_⁡*p*(*x*_*i*_)⌉ bits, where ⌈*r*⌉ denotes round up the number *r*. Therefore, the entropy *H*(*x*) of a discrete random variable *X* is defined as *H*(*X*) = −∑_*i*=0_^*N*−1^ *p*(*x*_*i*_) · log_2_ *p*(*x*_*i*_). High entropy is associated with a high variance in the pixel value, while low entropy indicates that the pixel values are fairly uniform.

#### 3.3.2. Local Two-Dimensional Information Entropy

Two-dimensional information entropy, which reflects the characteristics of spatial characteristics of gray-scale distribution, can effectively reflect the pixel neighborhood of space-related information. The two-dimensional entropy is obtained from a two-dimensional histogram, which is determined from the gray value of the pixels and the local average gray value of the pixels. For more details, one can refer to Sahoo and Arora [26].

#### 3.3.3. Local Haralick Texture Entropy

Haralick texture entropy [17], which is defined by a random variable, represents the size of the information of the image. Therefore, the quantitative measurement of the Haralick texture entropy can reflect iris texture. Haralick texture entropy is an intuitive descriptor of the texture structuredness in digital images. Shamir et al. [27] adopted Haralick texture entropy as an objective measurement that reflects the structural deterioration of the *C. elegans* muscle tissue during aging and achieved a good result. The Haralick texture entropy can be computed via

(5) $$\begin{array}{c} H\left(d,\theta \right)=-\sum_{i,j}p\left(i,j\;|\;d,\theta \right)\cdot \log p\left(i,j\;|\;d,\theta \right), \end{array}$$

where *i* and *j* are gray tone, *d* is the distance between two pixels, and *θ* is angle between two pixels. *p*(*i*, *j* | *d*, *θ*) is the relative frequencies of GLCM. Haralick texture entropy increase indicates that the subimage becomes less structured and more chaotic.

#### 3.3.4. Local Tamura Texture Measurement

Tamura et al. [28] pointed out that texture properties are defined for a region not for a point, and structure is defined as the repetitive patterns in which elements or primitives are arranged according to a placement rule, and it can be computed through equation *f* = *R*(*e*), where *R* denotes a placement rule and *e* denotes an element. Tamura developed six textural properties that are coarseness, contrast, directionality, line-likeness, regularity, and roughness, which correspond to human visual perception.

In this study, two texture elements proposed by Tamura are utilized, which are contrast and directionality. Contrast is a method of showing marked difference from stretching or shrinking of image gray scale and directionality reflects the shape and placement rule of the texture primitives.

The directionality can be computed with

(6) $$\begin{array}{c} F_{\text{dir}}=1-r\cdot n_{p}\cdot \sum_{p}^{n_{p}}\sum_{\phi \in w_{p}}{\left(\phi -\phi_{p}\right)}^{2}\cdot H_{D}\left(\phi \right), \end{array}$$

where *H*_*D*_ denotes the local direction histogram computed by Sobel edge operator, *n*_*p*_ denotes the number of peaks, *ϕ*_*p*_ denotes the *p*th peak position of *H*_*D*_, and *w*_*p*_ denotes the range of *p*th peak between valleys.

Set the variance and stand deviation of gray-level probability distribution to be *σ* ^2^ and *σ*; *μ*_4_ denotes the fourth moment of the mean; the kurtosis *α*_4_ is a well measure of polarization that can be defined as

(7) $$\begin{array}{c} \alpha_{4}=\frac{\mu_{4}}{\sigma_{4}}. \end{array}$$

This measure is normalized according to the range, so that it has the minimum value of one in the case of twin. Consequently, the contrast can be defined as

(8) $$\begin{array}{c} F_{\text{con}}=\frac{\sigma }{{\left(\alpha_{4}\right)}^{1/4}}. \end{array}$$

#### 3.3.5. Local Gray Variance

The gray variance reflects an important property of the image; the large variation values tend to yield at the location of edges [29]. It reflects the distribution uniformity of gray-scale; the more uneven the gray-scale distribution, the larger the variance. Let *f*(*i*, *j*) be the gray value of the pixel located at the point (*i*, *j*), a digital image *I* of size *M* × *N* pixels; set *E* to denote the mean of gray value, then *E* can be calculated as

(9) $$\begin{array}{c} E=\frac{\sum_{i=0}^{M-1}\sum_{j=0}^{N-1}f\left(i,j\right)}{M\times N}. \end{array}$$

Let *D* denote the variance of gray value, and then *D* can be calculated as

(10) $$\begin{array}{c} D=\sum_{i=0}^{M-1}\sum_{j=0}^{N-1}{\left(f\left(i,j\right)-E\right)}^{2}. \end{array}$$

**Figure 6:**
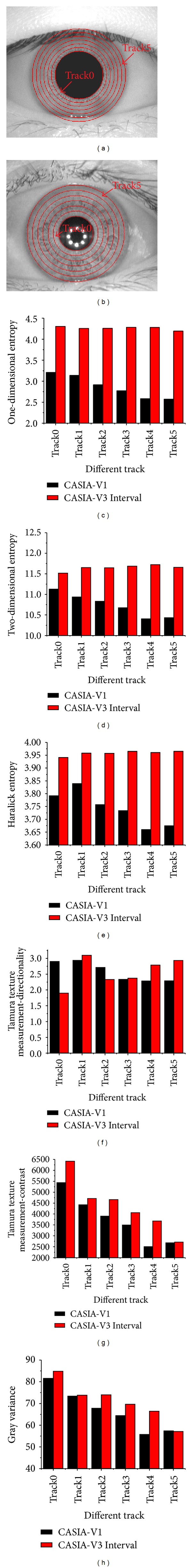
Bar chart of image quality evaluation. (a) CASIA-V1 image, (b) CASIA-V3 Interval image, (c) one-dimensional entropy, (d) two-dimensional entropy, (e) Haralick texture entropy, (f) Tamura texture directionality measurement, (g) Tamura texture contrast measurement, and (h) gray variance.

Set images of CASIA-V1 and CASIA-V3 Interval as experimental samples; the bar charts of these six evaluation parameters for different tracks are shown in Figure 6.

From Figures 6(a) and 6(b), it is observed that the iris texture of CASIA-V1 image is basically distributed in the inner ring near pupil, but the iris texture of CASIA-V3 Interval image is distributed relatively even, which means that tracks from track_0_ to track_5_ are full of clear iris texture. In view of these six quality evaluation parameters, as shown from Figure 6(c) to Figure 6(h), all of the six parameters' values of CASIA-V1 image render more distinctly decreasing trends, but only gray and contrast values of CASIA-V3 Interval image render decreasing trends. In the six tracks of CASIA-V3 Interval image, its ODE value, TDE value and HTE value are almost equal, and this result is consistent with the result of subjective quality evaluation.

In order to explain the problem more effectively, the subsets of the four databases are taken as samples to evaluate different tracks of images' properties. The percentages of maximum value of all the tracks are statistically calculated. The percentage distribution is shown in Table 2.

From Table 2, it can be seen that for CASIA-V1 database, the maximum values of all the six evaluation parameters mostly distributed close to the inner of iris ring, which include track0, track1, and track2. For CASIA-V3 database, the maximum values of the first four evaluation parameters are more likely to distribute in the middle ring, which include track1, track2, and track3, and the maximum values of the last two evaluation parameters distribute in both sides of iris ring. These results may be due to the inaccurate segmentation, which leads to iris ring containing eyelids and eyelashes, and further makes TMC and GV values abnormal. For MMU-V1 database, the maximum values of all the six evaluation parameters distribute in outer track. This is because its iris images have no clear texture in inner and middle tracks. For the JLUBRIRIS-V1 database, the maximum values of the last five evaluation parameters distribute in the middle or inner tracks, and the maximum value of ODE is distributed in outer track. The reason might be eyelashes disturbance.

From the above analysis, we can safely conclude that the six quality evaluation parameters show different characteristics on different iris databases. The following sections will discuss the combination of different quality evaluation factors.

### 3.4. Combining Different Quality Evaluation Factors

We focus on the different tracks of normalized iris image, mainly evaluate different tracks' texture information, and thus do not discard the iris image or different tracks completely.

Assuming that an iris database has *M* classes, each class has *N* images, and each normalized iris image is divided into *K* tracks. For convenience, ODE_*i*,*j*_^*k*^, TDE_*i*,*j*_^*k*^, HTE_*i*,*j*_^*k*^, TMD_*i*,*j*_^*k*^, TMC_*i*,*j*_^*k*^, and GV_*i*,*j*_^*k*^ denote labeled ODE, TDE, HTE, TMD, TMC, and GV, respectively, where subscript *i* denotes *i*th class, *j* denotes *j*th iris image, and superscript *k* denotes *k*th track of normalized segmented iris region.

#### 3.4.1. Different Quality Evaluation Parameter Vectorization

These six quality evaluation parameters are vectorized with the specific steps as follows.

**Step 1**

Processing all tracks in the iris database and getting six evaluation parameter values of every track.

**Step 2**

Forming one-dimensional vector. Every evaluation parameter is ranked firstly with the rule of track number, then class number and finally image number; therefore, six one-dimensional vectors are obtained, which are

(11) $$\begin{array}{c} \left\{{\text{ODE}}_{i,j}^{k}\;|\;\left(k\in \left[1,K\right],i\in \left[1,M\right],j\in \left[1,N\right]\right)\right\},\\ \left\{\text{TD}{\text{E}}_{i,j}^{k}\;|\;\left(k\in \left[1,K\right],i\in \left[1,M\right],j\in \left[1,N\right]\right)\right\},\\ \left\{\text{HT}{\text{E}}_{i,j}^{k}\;|\;\left(k\in \left[1,K\right],i\in \left[1,M\right],j\in \left[1,N\right]\right)\right\},\\ \left\{\text{TM}{\text{D}}_{i,j}^{k}\;|\;\left(k\in \left[1,K\right],i\in \left[1,M\right],j\in \left[1,N\right]\right)\right\},\\ \left\{\text{TM}{\text{C}}_{i,j}^{k}\;|\;\left(k\in \left[1,K\right],i\in \left[1,M\right],j\in \left[1,N\right]\right)\right\},\\ \left\{\text{G}{\text{V}}_{i,j}^{k}\;|\;\left(k\in \left[1,K\right],i\in \left[1,M\right],j\in \left[1,N\right]\right)\right\}. \end{array}$$

**Step 3**

Normalizing these six one-dimensional vectors and the normalization can be defined as

(12) $$\begin{array}{c} Nev=\frac{Oev-\min\left(ev\right)}{\max\left(ev\right)-\min\left(ev\right)}, \end{array}$$

where *Nev* represents normalized value, *Oev* represents raw value, and max⁡(*ev*) and min⁡(*ev*) represent the maximum and minimum values, respectively.

**Step 4**

Summing each normalized one-dimensional vector according to track number. Each one-dimensional vector has *K* values, which is the track number of every image. ODE can be calculated via

(13) $$\begin{array}{c} {\text{ODE}}^{k}=\sum_{i=1}^{M}\sum_{j=1}^{N}{\text{ODE}}_{i,j}^{k},\;k=0,\ldots ,K. \end{array}$$

The calculation processes of TDE, HTE, TMD, TMC and GV are similar to (13).

**Step 5**

Forming a two-dimensional vector. The result is shown in

(14) Track0Track1⋯TrackKODETDEHTETMDTMCGV[ODE0TDE0HTE0TMD0TMC0GV0ODE1TDE1HTE1TMD1TMC1GV1⋯⋯⋯⋯⋯⋯ODEKTDEKHTEKTMDKTMCKGVK].

#### 3.4.2. Measurement of Different Tracks' Feature Information with PSO

To generate an overall quality of iris images based on the estimated individual factors, an approach based on PSO algorithm is adopted to optimize different tracks' weighted coefficients according to quality evaluation results. PSO was first developed by Kennedy and Eberhart [30], which seeks to explore the search space by a population of particles.

To evaluate the improvement of performance achieved by the information fusion, the classical Daugman's measurement strategy [31] is adopted. As proposed by Daugman, for two-choice decisions (intra-class versus inter-class), the decidability index *d*′ measures how well the two types of distributions are separated, since recognition error corresponds to their overlap area, where *d*′ is defined as

(15) $$\begin{array}{c} d^{\prime }=\frac{\left|\mu_{1}-\mu_{2}\right|}{\sqrt{\left(\sigma_{1}^{2}+\sigma_{2}^{2}\right)/2\;}}, \end{array}$$

where *μ*_1_ and *μ*_2_ are the means of the two distributions and *σ*_1_ and *σ*_2_ are their standard deviations, respectively.

These six evaluation parameters are denoted as *w*_ODE_, *w*_TDE_, *w*_HTE_, *w*_TMD_, *w*_TMC_, and  *w*_GV_, respectively, and the their values are scaled in the range [0,1]. In the process of the PSO iterative optimization, the termination condition is the biggest *d*′ in a certain number of iterations. For convenience, our proposed algorithm is named as *PSO-QEW*, and there are six evaluation parameters in *PSO-QEW*.

Based on the obtained values of *w*_ODE_, *w*_TDE_, *w*_HTE_, *w*_TMD_, *w*_TMC_, and *w*_GV_, the weighted coefficients of different tracks are further calculated. For comprehensibility, the weighted coefficients of different tracks are denoted everywhere by *w* with a subscript *i*: *w*_*i*_ for track_*i*_, and *w*_*i*_ can be calculated via

(16) wi=(wODE·ODEi+wTDE·TDEi+wHTE·HTEi+wTMD·TMDi+wTMC·TMCi+wGV·GVi) ×(∑j=0K(wODE·ODEj+wTDE·TDEj+wHTE·HTEj+wTMD·TMDj+wTMC·TMCj+wGV·GVj))−1.

### 3.5. Fusing Texture Feature Information of Different Tracks

**Figure 7:**
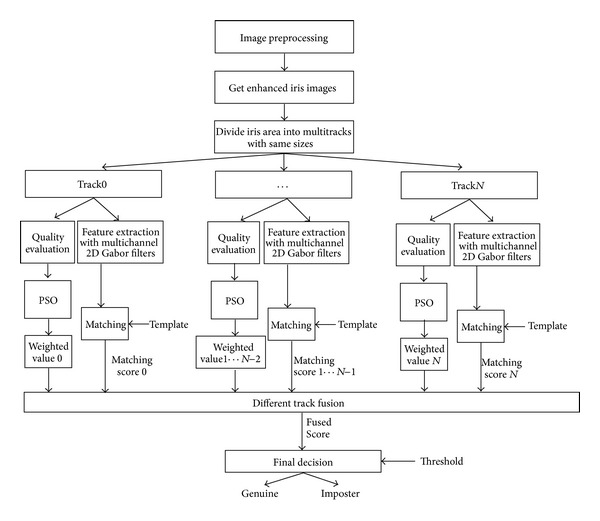
Architecture of the proposed fusion recognition system.

The iris recognition system based on feature fusion is designed in three steps. The first step is to divide the normalized segmented iris image into different number of tracks. The second step is to assign different weighted coefficients for each track. The third step is to adopt information fusion technology in iris recognition system. The architecture of the proposed fusion recognition system is shown in Figure 7.

## 4. Experiments Design

### 4.1. Description of Iris Image Databases

Public and free iris image database includes CASIA (four versions) [32] and MMU (two versions) [33]. CASIA database contains near infrared images and is by far the most widely used on iris biometric experiments. The CASIA-V1 database includes 756 iris image sequences from 108 subjects. The CASIA-V3 Interval database contains 2639 iris images from 395 different classes of 249 subjects, each iris image in this database is an 8-bit gray-level JPEG file with a resolution of 320 × 280 pixels. MMU-V1 iris database contributes a total number of 450 iris images, these iris images are contributed by 100 volunteers with different age, and nationalities. They come from Asia, Middle East, Africa, and Europe, each of them contributes 5 iris images for each eye.

**Figure 8:**
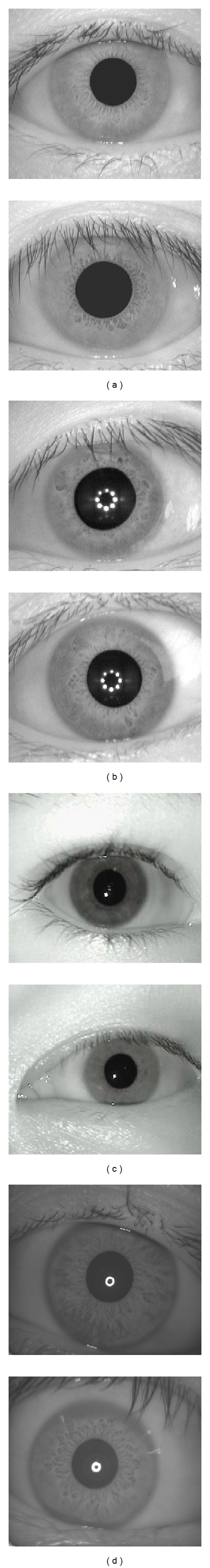
Sample images from CASIA-V1, CASIA-V3 Interval, MMU-V1, and JLUBRIRIS-V1 databases. (a) CASIA-V1, (b) CASIA-V3 Interval, (c) MMU-V1, and (d) JLUBRIRIS-V1.

JLUBRIRIS (two versions) [34] iris image database was established using self-developed iris image capture device and most of the iris images have enough texture information for reliable recognition. There are 180 different individuals in JLUBRIRIS-V1 iris image database; left and right iris images of each individual were captured under three different lighting conditions of 10 a.m., 14 p.m., and 19 p.m. and captured around 5s video image for each eye. The original image is 8-bit gray scale image with a size 480 × 576 pixels. The samples' year range is from nineteen to forty-five, and most of them are around the age of twenty, and the ratio of male to female is about eight to two. The samples of these four iris databases are shown in Figure 8.

From Figure 8, it can be seen that the iris images of different iris databases have different radii of pupil and limbic boundary; therefore, the normalized iris image is divided into different numbers of tracks according to distance between pupil and limbic boundary.

For comprehensibility, the radii of pupil and limbic boundary are denoted by *r*_*i*_ and *r*_*o*_, respectively, the distance between pupil and limbic boundary is denoted by *d*_*i*_, and the mean value of *d*_*i*_ is denoted by Mean(*d*_*i*_), then *d*_*i*_ = (*r*_*o*_ − *r*_*i*_). Assuming that one iris database has *N* images, so Mean(*d*_*i*_) = ∑(*d*_*i*_)/*N*. It can be seen that Mean(*d*_*i*_) is a statistical value. As shown in Table 3, four subsets databases are employed to obtain the Mean(*d*_*i*_) values, which of CASIA-V1, CASIA-V3 Interval, MMU-V1, and JLUBRIRIS-V1 are 48 pixels, 52 pixels, 27 pixels, and 107 pixels, respectively, and normalized iris is got via “Daugman Rubber Sheet” [14]. The sizes of normalized iris image of these four databases are 512 × 48 pixels, 512 × 48 pixels, 256 × 24 pixels and 512 × 64 pixels.

Let *I* be an iris track (*R* rows × *C* columns); in practical implementation, in order to accelerate computation speed, *R* and *C* are set to be *R* = 2^∧^ *n* and = 2^∧^ *n*  (*n* ∈ *Z*), where ^∧^ denotes power function. Set the height of each track be 8 pixels (*n* = 3), so the number of tracks for these four databases is 6, 6, 3, and 8, respectively.

### 4.2. Experiments Scheme and Setup

The proposed experimental framework is articulated through the following two major experiments.

1. The first experiment aims at analyzing the RAR on different regions of the iris. To realize this purpose, the normalized segmented iris region is divided into multitracks, and each track is estimated individually. Both GLCM and multichannel 2D Gabor filters are utilized to extract iris features; SVM and KNN are employed for classification.

For GLCM, in subsequent experiments, the window size is set at 8 × 8 pixels, gray levels at 256, and the distances between pixel pairs at 2 pixels according to our previous work. For multichannel 2D Gabor filters, in view of its symmetry character, six different direction values are set as *θ* = 0°, 30°, 60°, 90°, 120°, 150° and six values for 2, 4, 8, 16, 32, and 64 for the central frequencies, so they are 6 × 6 = 36 filters with different frequencies and directions. For SVM, the Grid-SVM employ 5-fold cross validation method and RBF kernel function. *C* is set at one and *γ* at 0.012. For KNN, the *k* is set at 2.

(2) The purpose of the second experiment is to fuse different tracks' information to improve the accuracy and robustness of iris recognition system. To achieve this purpose, local quality evaluation of different tracks is adopted. Meanwhile, PSO is employed to optimize these parameters' weighted coefficients and all tracks' information is fused according to different track's weighted coefficients.

For PSO, the acceleration coefficients *c*_1_ and *c*_2_ are set at 2, and the numbers of the iterations and particles are set at 50 and 5, respectively. According to our preliminary experiments, *w*_max⁡_ and *w*_min⁡_ are set at 0.9 and 0.4, respectively.

## 5. Experimental Results and Discussion

### 5.1. Experiment I—Analysis of Different Tracks' Information

In order to explain the effects of different tracks better, three groups of experiments, termed *TestGroupOne*, *TestGroupTwo*, and *TestGroupThree *are addressed, respectively. More details are as follows.

1. *TestGroupOne.* Take each track as experimental images, and take into account the effects of RAR for each track.
2. *TestGroupTwo.* Increase the size of experimental images gradually, and compare the RAR of overall normalized image and that of subimage.
3. *TestGroupThree.* From the overall normalized image, the size of experimental image decreased gradually. The RAR of overall normalized image is compared with that of subimage in another way.

All the tracks of these three groups of experiments are shown in Table 4. Labeled track0–*n* stands for the combination of tracks from number 0 to number *n*.

**Figure 9:**
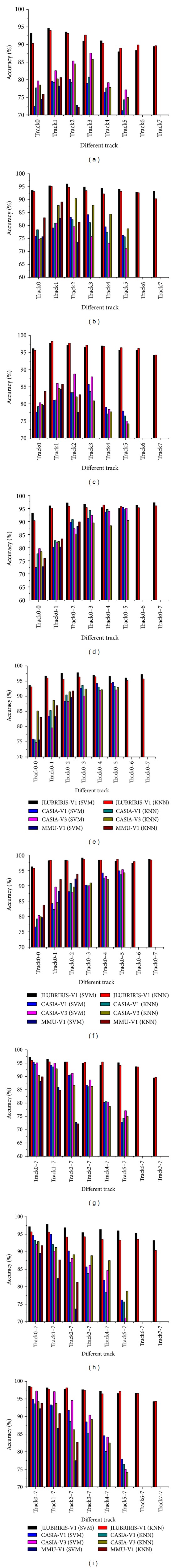
RAR of different tracks. (a) (b) (c) are experimental results of *TestGroupOne*, (a) is RAR produced by 2D Gabor features, (b) is RAR produced by GLCM features, and (c) is RAR produced by combined features. (d) (e) (f) are experimental results of *TestGroupTwo*, (d) is RAR produced by 2D Gabor features, (e) is RAR produced by GLCM features, and (f) is RAR produced by combined features. (h) (i) (j) are experimental results of *TestGroupThree*, (h) is RAR produced by 2D Gabor features, (i) is RAR produced by GLCM features, and (j) is RAR produced by combined features.

In *TestGroupOne*, *TestGroupTwo*, and *TestGroupThree* experiments, multichannel 2D Gabor filters and GLCM are adopted to extract iris features, and these two features are combined to form a new iris feature, named combined feature, therefore; we get three types of features. The experimental results are shown in Figure 9.

From Figure 9, it is observed that under the conditions of using the same feature extraction method and classifier, the RAR of different iris image databases has a certain difference. Among them, JLUBRIRIS-V1 has the highest RAR; MMU-V1 has the minimum RAR, and the RAR of CASIA-V1 and CASIA-V3 Interval is staggered, but remains consistent basically. The changing trend of JLUBRIRIS-V1 is smoother than that of the other three iris databases.

1. In *TestGroupOne.* Experimental results indicate that the middle tracks are slightly better than inner and outer tracks, and significant differences exist in the value of inner and middle tracks versus outer tracks. The middle tracks close to inner sides have higher RAR. Track1 and track2 of JLUBRIRIS-V1 have the highest RAR among all the eight tracks. Track2 and track3 of CASIA-V1 and CASIA-V3 Interval have the highest RAR among all the six tracks. Track1 of MMU-V1 has the highest RAR among the three tracks.
2. In *TestGroupTwo.* With the size of iris subregion increasing, its RAR also increases gradually and is more stable. In JLUBRIRIS-V1, there is a phenomenon that the RAR of track0–2 or track0–3 is slightly higher than that of track0–7. However, compared with that of the other three iris databases, this phenomenon is not evident. As in CASIA-V1 and CASIA-V3 Interval, the RAR of track0–4 is close to but still smaller than that of track0–5, and in MMU-V1, the RAR of track0-0 and track0-1 is less than that of track0–2.
3. In *TestGroupThree.* RAR also gradually decreases with the size of iris subregion decreasing. In all the experimental iris databases, only the RAR of track1–7 of JLUBRIRIS-V1 is slightly higher than that of track0–7, while the decreasing trend of RAR is more apparent with the size of iris regions reducing in the three other iris databases. This phenomenon also further exactly verifies that inner and middle tracks close to pupil play an important role in iris recognition. Nevertheless, partial iris region cannot completely replace the entire iris region. Therefore, different tracks that take different weighted coefficients have practical significance.

Based on the three groups of experimental results above, we can safely draw conclusions as follows.

1. Different iris regions have different RAR. This means that each track has different feature information; several tracks close to pupil contain more feature information, but the information contained in the tracks close to sclera shows a gradually decreasing trend.
2. Track0 has lower RAR than that of the middle tracks, and the reason might be the inaccurate iris segmentation, which causes inconsistency and results in the track0 to be affected by pupil boundary and further causes a certain effect on RAR.
3. Partial iris image may not completely replace the entire iris image for iris recognition in a way. Although some tracks have low RAR, this does not mean these tracks do not contribute to the accuracy of the entire iris region; it simply means there are inconsistencies or fragile bits to some degree.

### 5.2. Experiment II—Effect of Fusion Different Tracks' Information

#### 5.2.1. Empirical Evidence of Improved Decidability

For iris recognition, selecting an appropriate similarity measurement for matching feature vectors is essential. There are many measurements such as Hamming distance, weighted Hamming distance, mean of Euclidean distance [35], cosine similarity, and weighted AND-NOT distance. In this study, traditional Euclidean distance (ED) measurement is adopted as the matching criterion, and ED can be calculated via

(17) $$\begin{array}{c} \text{ED}=\sqrt{\sum_{i=1}^{M}{\left({\text{Template}}_{i}-{\text{Test}}_{i}\right)}^{2}}, \end{array}$$

where *M* is the dimension of the feature vector, Template_*i*_ is the *i*th component of template sample feature vector, and Test_*i*_ is the *i*th component of test sample feature vector.

For each iris database, part of images are set as template images, the other part of images as test images; each test image is individually matched to the other entire template images and get the corresponding ED. ED obtained by the same class image is named as intra-class ED, and ED obtained by the nonsame class image is named as inter-class ED. In the subsequent experiments, the entire normalized iris and different tracks need matching, respectively, form a total of twenty-seven groups (seven groups for CASIA-V1, seven groups for CASIA-V3 Interval, four groups for MMU-V1, and nine groups for JLUBRIRIS-V1).

**Figure 10:**
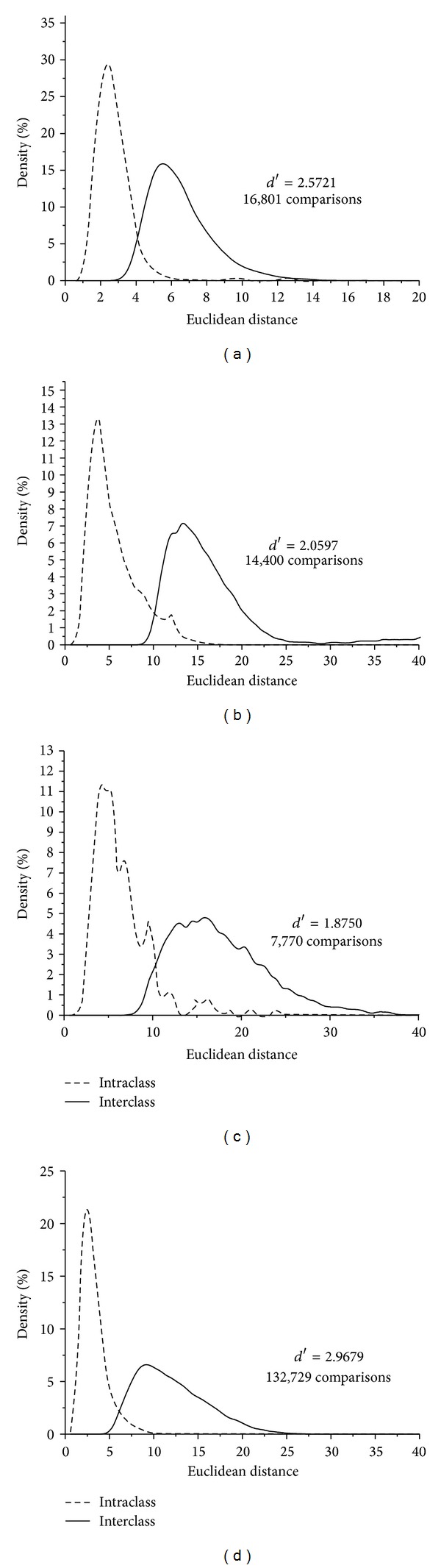
Distance distributions of the intra-class and inter-class patterns for the four iris image databases without fusion. (a) CAISA-V1, (b) CASIA-V3 Interval, (c) MMU-V1 and (d) JLUBRIRIS-V1 iris image databases.

By measuring separation, the performance of iris recognition system can be calibrated by *d*′ score. The greater the *d*′ value is, the more separable the two distributions become, and the lower *d*′ value indicates less robust. For entire normalized iris, the distributions of intra-class ED and inter-class ED are illustrated in Figure 10.

The *d*′ values of different tracks are shown in Table 5. After local quality evaluation and PSO optimum operation, weighted coefficients of quality evaluation parameters are shown in Table 6. According to (16), different tracks' weighted coefficients are further obtained which are shown in Table 7.

From Table 5, it can be seen that middle tracks have higher *d*′ values, and outer track has lower *d*′ values which indicates that the middle tracks are more separable and further indicates that middle tracks close to pupil have more information. From Table 6, it can be seen that six quality parameters' weighted coefficients are different for four different iris databases. It is likely to conclude that the proposed *PSO-QEW* is a self-adaptive quality evaluation algorithm, and it can automatically optimize parameters according to the different databases' characteristics. From Table 7, we may see that middle tracks have higher weighted coefficients and the outer tracks have lower weighted coefficient, which is consistent with the conclusions of Section 5.1.

**Figure 11:**
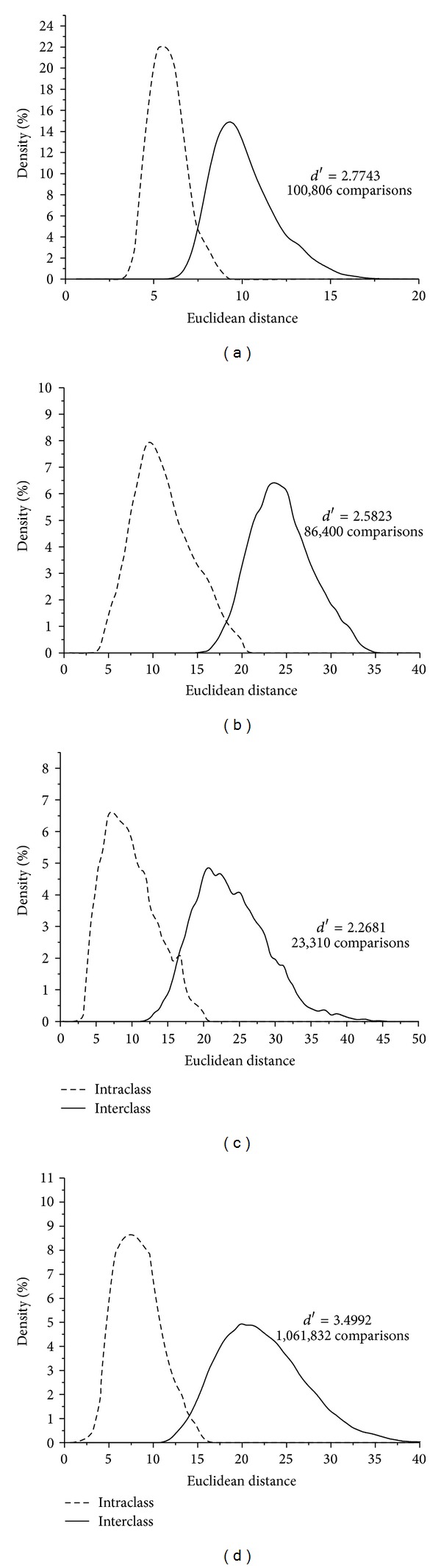
Distance distributions of the intra-class and inter-class patterns for the four iris image databases with fusion. (a) CAISA-V1, (b) CASIA-V3 Interval, (c) MMU-V1 and (d) JLUBRIRIS-V1.

After fusion of each track, the distributions of intra-class ED and inter-class ED are illustrated in Figure 11.

As can be seen from Figures 10 and 11, we can see that *d*′ values are improved after fusing different tracks' texture feature information. In Figure 10, the *d*′ values of CASIA-V1, CASIA-V3 Interval, MMU-V1, and JLUBRIRIS-V1 are 2.5721, 2.0597, 1.8750, and 2.9679, respectively. However, in Figure 11, the *d*′ values can increase to 2.7743, 2.5823, 2.2681, and 3.4992 after multitracks fusion.

#### 5.2.2. Empirical Evidence of Lowered EER

The proposed algorithm is evaluated by false accept rate (FAR), false reject rate (FRR), receiver operating characteristic (ROC) curve and equal error rate (ERR). The FAR is the probability of accepting an imposter as an authorized subject and FRR is the probability of an authorized subject being incorrectly rejected. The ROC curve is used to report the performance of the proposed method [9]. Cross-point of FAR and FRR is ERR, and the lower the ERR is, the better the algorithm is.

**Figure 12:**
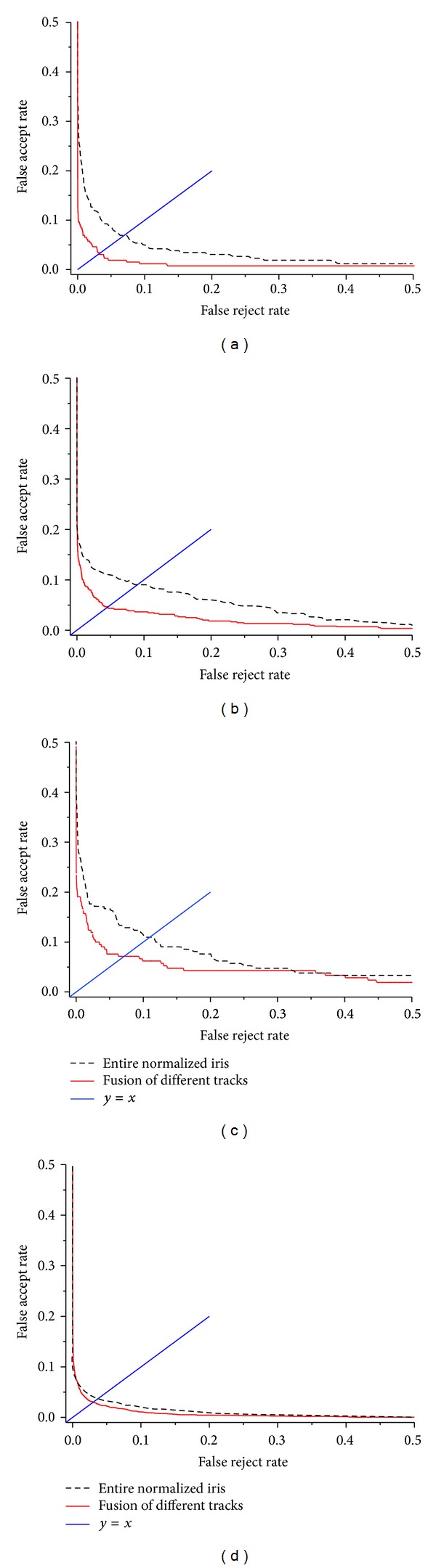
ROC curves for (a) CASIA-V1, (b) CASIA-V3 Interval, (c) MMU-V1 and (d) JLUBRIRIS-V1 databases.

From Figure 12, it is can be seen that EER of fused different tracks is less than that of entire normalized iris, and this indicates that our fusion algorithm is effective.

Each track's EER value is shown in Table 8. From Table 8, two conclusions are drawn. (1) The EER of each track is greater than that of entire normalized iris, and this proves again that partial iris may not simply replace the entire iris in a way. (2) The EER of entire normalized iris is greater than that of fused different tracks, and it indicates that proposed fusion scheme can effectively improve the performance of iris recognition system.

In sum, it is likely to conclude that the multitrack feature fusion can make the distributions of intra-class and inter-class more separable and make iris recognition system achieve more robust performance.

### 5.3. Comparison and Discussion

We analyze the characteristics of different regions of iris with multiple perspectives, and the experimental results keep consistent conclusion basically. The tracks with rich texture information can achieve higher RAR. However, it cannot simply use partial image instead of the entire image for, at least, two reasons. (1) Only selecting partial image will lose some information; (2) fragile bits exist in the iris image. Hollingsworth et al. [5, 8, 36, 37], Bolle et al. [7], and Dozier et al. [38, 39] have proved the existence of the fragile bits from some aspects, and fragile bits also influence RAR to a certain extent. For comparison purpose, other methods are listed in Table 9.

Compared to directly processing the entire normalized iris image, the fusion method effectively reduces fragile bits' effect on reliability and accuracy of recognition system. The experimental results in the last two sections show that, either EER or *d*′ values, achieved by fusion of different tracks, are greatly superior to those by unfused different tracks.

## 6. Conclusion and Future Work

We propose a feature information fusion scheme for different portions of iris on the basis of local quality evaluation. We firstly analyze the different iris subregions' influence on RAR, and then adopt six local quality evaluation methods to analyze different subregions' texture information and finally fuse different subregions' information according to corresponding weighted coefficients. Three public accessible databases and a private database are used in our experiments, which are (1) CASIA-V1; (2) CASIA-V3 Interval; (3) MMU-V1; and (4) JLUBRIRIS-V1 databases. These databases cover a wide range of iris image types. The experimental results demonstrate that our fusion algorithm can effectively improve the performance of iris recognition system.

More attention will be paid to evaluating the proposed system in more other iris image databases. In addition, we will continuously focus on investigation of a flexible iris segmentation method and more flexible fusion strategy.

## Figures and Tables

**Table 1:** Comparison of different methods.

Methods	Iris database	Feature extraction methods	Evaluation methods	Conclusions
Du et al. [3]	CASIA-V1	1-D Log Gabor	Accuracy rate	Inner rings of iris have more distinguishable and individually unique signal, and a partial iris image can be used for human identification

Hollingsworth et al. [5]	ICE (NIR)	1-D Log Gabor, 2D Gabor	Hamming distance	Difference in consistency of iris code bits based on the size of filter used. The middle bands may be slightly better than either the inner or the outer bands

Pereira and Veiga [6]	CASIA-V1	Genetic algorithm	Hamming distance	The points that were selected non-uniformly over the iris region are more reliable than those selected uniformly

Bolle et al. [7]	—	—	—	Explained that not all bits are equally to flip but some bits are fragile bits

Yuan and Shi [11]	—	Quadrature Log-Gabor	Hamming distance	Inner has abundance of minute texture, middle has bigger block of texture, and outer is the flattest of the iris texture

Broussard et al. [1]	UBIRIS	Directional energy filter	Accuracy rate	About 40 percent of the iris produced the highest accuracy; the left and right iris produce higher identification accuracy than the top and bottom iris

Poursaberi and Araabi [13]	CASIA-V1	Wavelet Daubechies2	Hamming and harmonic mean distance	Observed that relying on a smaller but more reliable part of the iris can improve the overall performance

Proposed	CASIA-V1, CASIA-V3 Interval, MMU-V1, JLUBRIRIS-V1	2D Gabor, GLCM	SVM, KNN,	Different iris regions on the recognition accuracy will produce different effects; several tracks close to pupil contain more feature information; track0 has much less identification accuracy than the middle tracks do; partial iris can not completely replace the entire iris

**Table 2:** Percentage distribution of maximum values.

Iris databases	Track number	Percentage of maximum values (%)					
		ODE	TDE	HTE	TMD	TMC	GV
CASIA-V1	Track 0	46.92	30.38	9.89	50.00	91.63	91.54
	Track 1	29.23	24.62	26.62	29.62	0.00	0.38
	Track 2	12.69	33.85	33.46	13.46	0.38	0.38
	Track 3	6.15	6.15	20.53	2.69	2.28	2.31
	Track 4	2.69	3.46	4.94	3.46	1.53	1.54
	Track 5	2.32	1.54	4.56	0.77	4.18	3.85

CASIA-V3 Interval	Track 0	12.08	0.86	47.08	0.00	32.08	33.33
	Track 1	16.66	56.66	18.75	17.50	5.41	5.83
	Track 2	7.08	7.08	7.92	45.41	4.16	4.17
	Track 3	29.58	20.41	12.50	22.5	10.00	8.33
	Track 4	27.08	9.58	8.75	5.41	9.58	9.58
	Track 5	7.52	5.41	5.00	9.18	38.77	38.76

MMU-V1	Track 0	27.93	18.99	15.08	0.00	15.64	15.64
	Track 1	2.23	46.37	31.84	24.58	22.91	20.11
	Track 2	69.84	34.64	53.08	75.42	61.45	64.25

JLUBRIRIS-V1	Track 0	24.97	67.63	75.45	0.00	30.32	31.41
	Track 1	0.00	16.74	5.21	15.50	5.90	2.47
	Track 2	0.00	8.09	9.47	40.74	0.79	1.37
	Track 3	0.55	4.66	2.47	31.28	4.94	4.39
	Track 4	2.74	2.06	0.55	6.86	8.94	9.19
	Track 5	3.16	0.27	1.37	3.16	17.97	17.01
	Track 6	16.32	0.14	1.78	2.19	14.54	15.50
	Track 7	52.26	0.41	3.70	0.27	16.60	18.66

**Table 3:** Properties of different iris databases.

Iris databases	No. of subclasses	No. of images	Mean (*d*_i_)	Size of normalized iris	No. of tracks
CASIA-V1	65	260	48 pixels	512 × 48 pixels	6
CASIA-V3 Interval	24	240	52 pixels	512 × 48 pixels	6
MMU-V1	37	179	27 pixels	256 × 24 pixels	3
JLUBRIRIS-V1	50	729	107 pixels	512 × 64 pixels	8

**Table 4:** Description of the three groups of experiments.

Group of experiments	Experimental subimages			
	CASIA-V1	CASIA-V3 Interval	MMU-V1	JLUBRIRIS-V1
*Testgroupone *	Track 0, track 1, track 2, track 3, track 4, track 5	Track 0, track 1, track 2, track 3, track 4, track 5	Track 0, track 1, track 2	Track 0, track 1, track 2, track 3, track 4, track 5, track 6, track 7

*Testgrouptwo *	Track 0-0, track 0-1, track 0–2, track 0–3, track 0–4, track 0–5	Track 0-0, track 0-1, track 0–2, track 0–3, track 0–4, track 0–5	Track 0-0, track 0-1, track 0–2	Track 0-0, track 0-1, track 0–2, track 0–3, track 0–4, track 0–5, track 0–6, track 0–7

*Testgroupthree *	Track 0–5, track 1–5, track 2–5, track 3–5, track 4-5, track 5-5	Track 0–5, track 1–5, track 2–5, track 3–5, track 4-5, track 5-5	Track 0–2, track 1-2, track 2-2	Track 0–7, track 1–7, track 2–7, track 3–7, track 4–7, track 5–7, track 6-7, track 7-7

**Table 5:** *d*′ values of different tracks.

Track number	*d*′ values			
	CASIA-V1	CASIA-V3 Interval	MMU-V1	JLUBRISI-V1
Track 0	0.9913	1.5281	1.8682	1.9088
Track 1	1.7394	1.9793	1.7604	2.2657
Track 2	1.8912	2.1174	1.6977	2.2691
Track 3	1.8141	1.6560	—	2.1238
Track 4	1.8898	1.6820	—	1.9651
Track 5	1.7223	1.6157	—	1.8367
Track 6	—	—	—	1.8651
Track 7	—	—	—	1.5476

**Table 6:** Weighted coefficients of quality evaluation parameters.

Quality evaluation parameters	Weighted coefficients			
	CASIA-V1	CASIA-V3 Interval	MMU-V1	JLUBRISI-V1
ODE	0.000	0.500	0.394	0.031
TDE	0.000	0.500	0.606	0.181
HTE	1.000	0.000	0.000	0.275
TMD	0.000	0.000	0.000	0.104
TMC	0.000	0.000	0.000	0.121
GV	0.000	0.000	0.000	0.288

**Table 7:** Weighted coefficients of different tracks.

Track number	Weighted coefficients			
	CASIA-V1	CASIA-V3 Interval	MMU-V1	JLUBRIRIS-V1
Track 0	0.152426	0.147151	0.314537	0.134488
Track 1	0.173540	0.175048	0.330402	0.124607
Track 2	0.174413	0.169740	0.355061	0.123017
Track 3	0.170651	0.175408	—	0.127094
Track 4	0.165613	0.172419	—	0.127358
Track 5	0.163357	0.160233	—	0.126319
Track 6	—	—	—	0.121973
Track 7	—	—	—	0.115144

**Table 8:** EER of different tracks.

Track number	EER values			
	CASIA-V1	CASIA-V3 Interval	MMU-V1	JLUBRIRIS-V1
Track 0	0.30679	0.21486	0.17619	0.13927
Track 1	0.15337	0.13333	0.18003	0.09857
Track 2	0.14615	0.10833	0.19520	0.09012
Track 3	0.14573	0.17500	—	0.11416
Track 4	0.15000	0.17333	—	0.13268
Track 5	0.16911	0.18833	—	0.15982
Track 6	—	—	—	0.15724
Track 7	—	—	—	0.21035
Entire normalized iris	0.06923	0.09000	0.10952	0.03747
Fuse different tracks	0.03239	0.04551	0.07169	0.02982

**Table 9:** Description of process fragile bits.

Methodology	Process method of fragile bits	Results
Hollingsworth et al. [5, 36]	Masking iris code bits corresponding to complex filter response near the axes of the complex plane	Improve the separation between the match and nonmatch Hamming distance distributions

Hollingsworth et al. [8]	Consider the fragile bits provide beneficial information rather than ignoring fragile bits completely	Score fusion of fragile bit distance and Hamming distance works better than Hamming alone, and it improved the accuracy of matches

Hollingsworth et al. [37]	Masks fragile bits	*d*′ value is 8.2516 compared with 7.4825 for nonmask fragile bits

Bolle et al. [7]	Theoretically proved the existence of fragile bits	—

Dozier et al. [38]	Used genetic search to minimize the number of iris code bits	Approximately reduce 89% of iris code bits and discard the fragile bits

Dozier et al. [39]	Only kept bits that were 90% or 100% consistent	Discard the fragile bits

Proposed	Divided normalized iris into multitracks, and fused all tracks by local quality evaluation with PSO	*d*′ value increase and EER decrease
